# Inhibiting signal transducer and activator of transcription-3 increases response to gemcitabine and delays progression of pancreatic cancer

**DOI:** 10.1186/1476-4598-12-104

**Published:** 2013-09-11

**Authors:** Kolaparthi Venkatasubbarao, Lindsay Peterson, Shujie Zhao, Ping Hill, Lin Cao, Qing Zhou, Steffan T Nawrocki, James W Freeman

**Affiliations:** 1 Department of Medicine, Division of Hematology and Oncology, University of Texas Health Science Center at San Antonio, 7703 Floyd Curl Drive, San Antonio, TX 78229-3900, USA; 2 Cancer Therapy and Research Center, Experimental and Developmental Therapeutics Program, 7979 Wurzbach Rd, San Antonio, TX 78229, USA; 3 Research and Development, Audie Murphy Veterans Administration Hospital, San Antonio, TX 78229, USA

**Keywords:** Pancreatic cancer, STAT3, EGFR inhibitor, Cancer therapy

## Abstract

**Background:**

Among the solid tumors, human pancreatic ductal adenocarcinoma (PDAC) has the worst prognosis. Gemcitabine is the standard first line of therapy for pancreatic cancer but has limited efficacy due to inherent or rapid development of resistance and combining EGFR inhibitors with this regimen results in only a modest clinical benefit. The goal of this study was to identify molecular targets that are activated during gemcitabine therapy alone or in combination with an EGFR inhibitor.

**Methods:**

PDAC cell lines were used to determine molecular changes and rates of growth after treatment with gemcitabine or an EGFR inhibitor, AG1478, by Western blot analysis and MTT assays respectively. Flow cytometric analysis was performed to study the cell cycle progression and rate of apoptosis after gemcitabine treatment. ShRNA was used to knockdown STAT3. An *in vivo* orthotopic animal model was used to evaluate STAT3 as a target. Immunohistochemical analysis was performed to analyze Ki67 and STAT3 expression in tumors.

**Results:**

Treatment with gemcitabine increased the levels of EGFR^Tyr1068^ and ERK phosphorylation in the PDAC cell lines tested. The constitutive STAT3^Tyr705^ phosphorylation observed in PDAC cell lines was not altered by treatment with gemcitabine. Treatment of cells with gemcitabine or AG1478 resulted in differential rate of growth inhibition. AG1478 efficiently blocked the phosphorylation of EGFR^Tyr1068^ and inhibited the phosphorylation of down-stream effectors AKT and ERKs, while STAT3^Tyr705^ phosphorylation remained unchanged. Combining these two agents neither induced synergistic growth suppression nor inhibited STAT3^Tyr705^ phosphorylation, thus prompting further studies to assess whether targeting STAT3 improves the response to gemcitabine or AG1478. Indeed, knockdown of STAT3 increased sensitivity to gemcitabine by inducing pro-apoptotic signals and by increasing G1 cell cycle arrest. However, knockdown of STAT3 did not enhance the growth inhibitory potential of AG1478. *In vivo* orthotopic animal model results show that knockdown of STAT3 caused a significant reduction in tumor burden and delayed tumor progression with increased response to gemcitabine associated with a decrease in the Ki-67 positive cells.

**Conclusions:**

This study suggests that STAT3 should be considered an important molecular target for therapy of PDAC for enhancing the response to gemcitabine.

## Background

PDAC is the fourth leading cause of cancer deaths in the United States and has the worst prognosis of all solid tumors. This year alone it is estimated that 38,460 of the 45,220 patients diagnosed with pancreatic cancer in the United States will succumb to the disease [1]. Despite advancements in the understanding of the genetics of this disease and the use of combined chemotherapy and targeted biological agents, the management of this lethal malignancy remains one of the greatest oncological challenges [2].

At the time of clinical presentation, most PDAC patients have advanced disease, either locally or with distant metastasis. Diagnosis at this late stage is likely due to the absence of specific early signs and symptoms of disease and the lack of reliable screening tests that would allow for therapy at an earlier, potentially curable stage [2]. Less than 20% of patients are diagnosed with disease that is amenable for surgical intervention [3]. Sadly, about half of all patients with this disease die within the first six months of diagnosis resulting in a five-year survival rate of less than 5% [2].

The most striking genetic signature of PDAC is mutations of codon-12 of the *c-K-Ras* gene and inactivating mutations of *INK4a,* which occur in greater than 90% of pancreatic tumors [4]. More than half of PDAC tumors also exhibit loss of the functional tumor suppressor gene, *deleted in pancreatic cancer, locus 4* (*DPC4*), either due to homozygous deletion or intragenic mutations, and up to 75% of PDAC have a *p53* mutation [4]. As found with other solid tumors, PDAC shows aberrant over-expression and/or constitutive activation of a number of growth factor receptors [5].

In 1997, Burris et al. [6] showed a survival benefit for patients treated with gemcitabine compared with 5-fluorouracil and since that time gemcitabine has been the most used first-line therapy for the management of PDAC [7]. The clinical response rate of PDAC to gemcitabine is less than 25% and those tumors that show an initial response generally develop resistance during the course of therapy [8,9]. The rapid development of resistance to gemcitabine may be mediated either by molecular changes of tumor cells or due to selection of a pre-existing sub-population of tumor cells that are inherently resistant to chemotherapy. There continue to be clinical trials that use gemcitabine in combination with other chemotherapeutic or biologic targeted agents. Erlotinib, an EGFR kinase inhibitor, in combination with gemcitabine was approved as therapy for PDAC on the basis of a survival benefit of approximately two weeks [10]. However, the enthusiasm for the addition of erlotinib is dampened because of the high cost, minimal increase in survival benefit, prevalence of *K-Ras* mutations in most PDAC, and the potential for additional toxicity. Recent studies [11,12] show that FOLFIRINOX (5-fluorouracil, leucovorin, irinotecan, and oxaliplatin) provides a short-term survival benefit over gemcitabine; however, this regimen is restricted to patients that have a good functional status. Thus, new therapeutic targets and approaches are being sought to further improve the survival of patients with PDAC.

Signal transducer and activation of transcription (STAT) is a family of transcription factors known to mediate cytokine and growth factor responses in a wide variety of cells [13]. Among these proteins, STAT3 is often constitutively activated and contributes to tumor progression and resistance to apoptosis in both solid and hematological malignancies [13,14]. We previously found that STAT3 was constitutively activated in PDAC [15] and it plays a role in the maintenance of a cancer stem cell phenotype [16,17].

This study investigated whether STAT3 may be an independent therapeutic target or may enhance response to gemcitabine. *In vitro* studies show that constitutive STAT3^Tyr705^ phosphorylation is not prevented by inhibiting EGFR activation with an EGFR kinase inhibitor (AG1478) or by treating cells with gemcitabine. Knocking down STAT3 enhanced gemcitabine induced growth inhibition *in vitro* by increasing G1 cell cycle arrest and pro-apoptotic signals. Studies using an *in vivo* orthotopic mouse model showed that knocking down STAT3 (BxPC3/shSTAT3) delayed tumor progression and increased sensitivity to gemcitabine supporting the *in vitro* findings that STAT3 may be a relevant target for improving therapeutic responses.

## Results

### Constitutive STAT3^Tyr705^ phosphorylation remains relatively unchanged after gemcitabine treatment while EGFR^Tyr1068^ and ERK phosphorylation is increased

**Figure 1:**
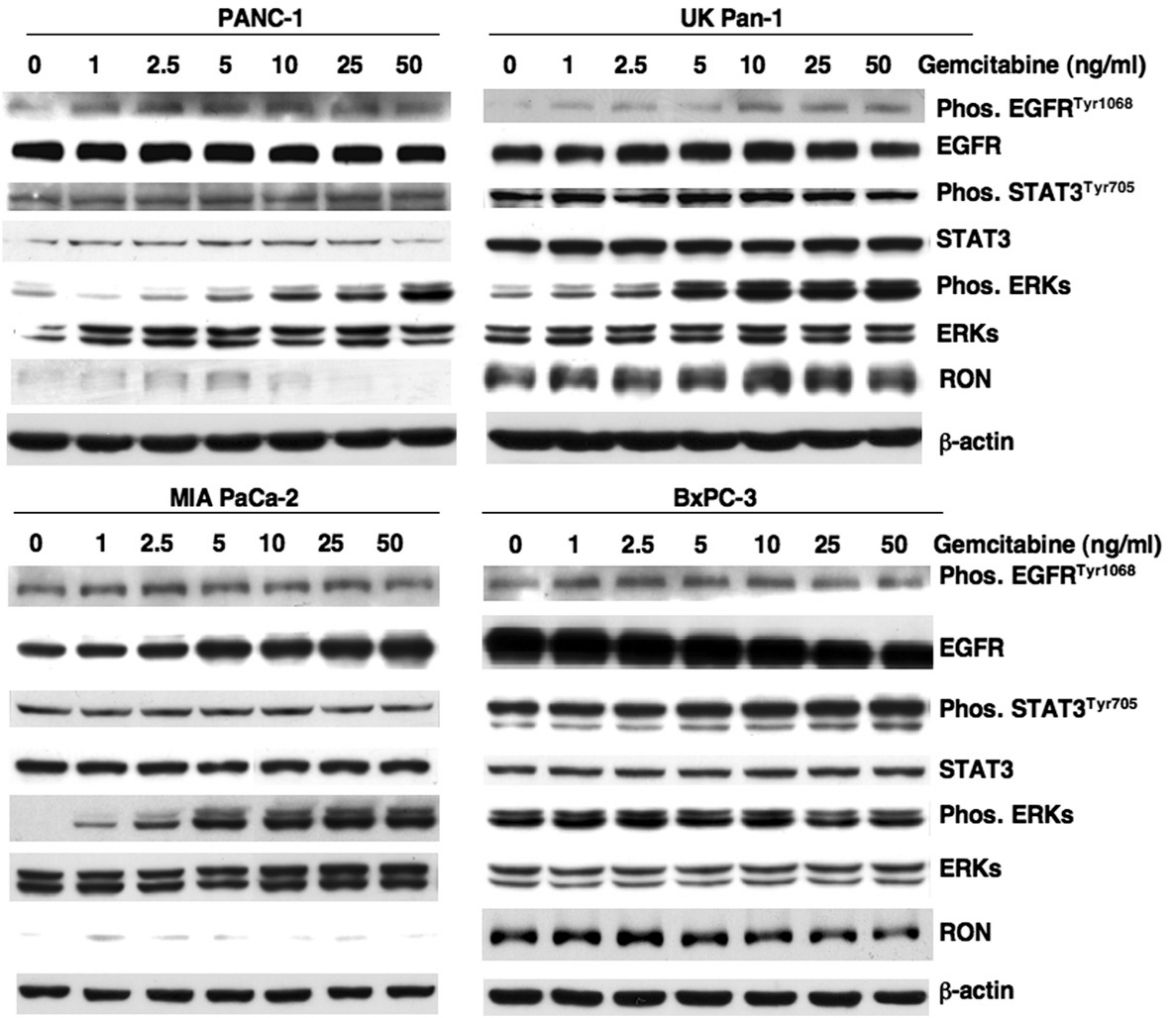
**Constitutive STAT3**^**Tyr705 **^**phosphorylation is not inhibited by gemcitabine in PDAC cells.** Exponentially growing PDAC cells were treated with indicated concentrations of gemcitabine for 96 hrs and cellular lysates were subjected to Western blot analysis. The blots were probed for indicated phospho-specific proteins and their respective total forms. As a loading control, human β-actin levels are shown.

The effects of gemcitabine on the phosphorylation levels of EGFR, STAT3, and ERKs were determined in four PDAC cell lines. PANC-1, UK Pan-1, MIA PaCa-2 and BxPC3 cells were treated with increasing doses of gemcitabine for 96 h and total cellular lysates were analyzed by Western blots (Figure 1). EGFR^Tyr1068^ phosphorylation was modestly increased after gemcitabine treatment although the levels of STAT3^Tyr705^ phosphorylation were relatively constant for all doses used. Phosphorylation of ERKs was also increased in a dose-dependent manner in three of the cell lines (PANC-1, UK-Pan-1, MIA PaCa-2); whereas, ERKs were constitutively phosphorylated in BxPC3 cells (Figure 1).

RON receptor kinase is a member of the c-Met family and is reported to play a role in PDAC carcinogenesis. Previous studies demonstrated that RON plays a role in resistance to gemcitabine and suppression of RON inhibited the expression of STAT3^Tyr705^[18]. The four cell lines examined in this study showed different expression levels of RON suggesting STAT3 expression and its phosphorylation (Figure 1) is independent of RON expression in some PDAC cells. Moreover, RON expression was not appreciably changed by treatment with gemcitabine (Figure 1).

### EGFR inhibitor AG1478 differentially inhibited the growth of PDAC cells while constitutive STAT3^Tyr705^ phosphorylation is not affected

**Figure 2:**
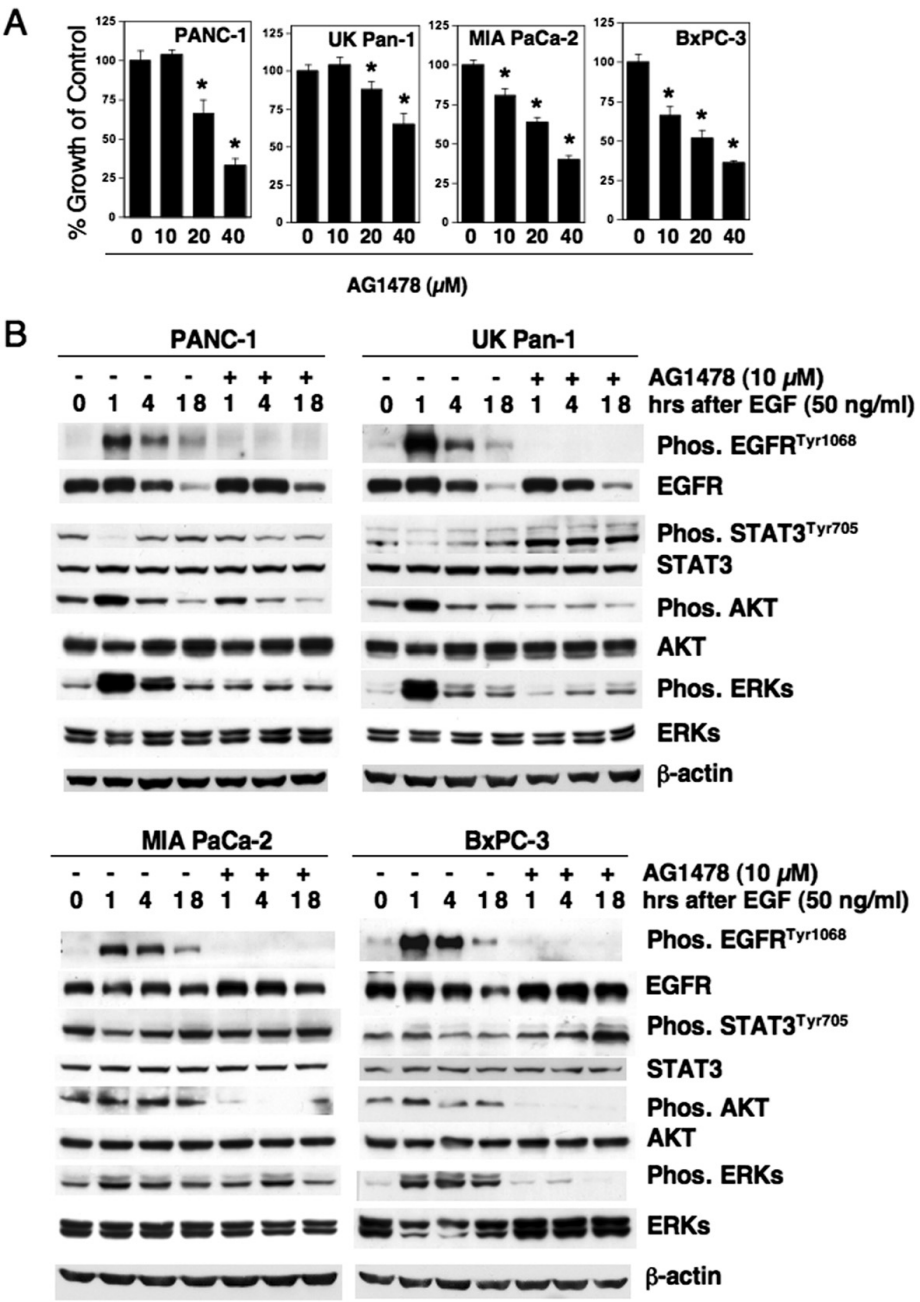
**EGFR inhibitor AG1478 suppresses growth of human pancreatic cancer cells *in vitro *and constitutive STAT3**^**Tyr705 **^**phosphorylation is not suppressed by EGFR inhibitor AG1478 in PDAC cells. A**, Exponentially growing PDAC cells were treated with indicated concentrations of EGFR inhibitor AG1478 for 96 h and MTT assays were performed to measure cell growth. Data are presented as % growth of control. Bars represent SD of eight replicates, and experiments repeated for three times. (*) = highly significant with a p < 0.001. **B**, PDAC cells were serum deprived for 48 h and pre-treated with EGFR inhibitor, AG1478 (10 μM) for 4 h followed by stimulation with EGF (50 ng/mL) for indicated periods of time in the presence or absence of EGFR inhibitor. Vehicle DMSO was used where EGFR inhibitor was not used. Equal amounts of proteins were loaded and the blots were probed for phosphorylated and total forms of EGFR, STAT3, AKT and ERKs. As a loading control, human β-actin levels are shown.

The ErbB family member EGFR is over-expressed and shows hyperactivity in many tumor types, including PDAC, and is recognized as an important molecular target for therapy. This aberrant activity of EGFR or other ErbB family members activate a number of down stream targets and may contribute to the constitutive STAT3^Tyr705^ phosphorylation found in cancer cells [15]. Hyperactivity of EGFR or other growth factor pathways is also thought to play a role in resistance to gemcitabine [19]. We evaluated the effect of an EGFR inhibitor, AG1478, on the growth of PDAC cell lines, PANC-1, UK Pan-1, MIA PaCa-2 and BxPC3. AG1478 inhibited cell growth of the four PDAC cell lines in a dose dependent manner; although, UK Pan-1 was less sensitive compared to the other three cell lines (Figure 2A). Only MIA PaCa-2 and BxPC3 cells showed significant growth inhibition at 10 μM concentration of AG1478, that was sufficient enough to inhibit the phosphorylation of EGFR^Tyr1068^ in all four cell lines tested (Figure 2B). Significant inhibition of growth of UK Pan-1 with AG1478 required concentrations of 20 μM or higher doses which are greater than that required for inhibiting phosphorylation of EGFR^Tyr1068^. This raises the possibility that this growth inhibition may not be specific in regards to inhibiting EGFR signaling (Figure 2A,B).

In order to determine the effect of AG1478 on the phosphorylation of EGFR and potential down stream signaling targets including STAT3, cell lines were stimulated with EGF. Stimulation with EGF induced a robust, but transient increase of EGFR^Tyr1068^ phosphorylation, as well as phosphorylation of down stream targets AKT and ERKs in all of the cell lines tested (Figure 2B). In cells treated with AG1478, EGFR^Tyr1068^ phosphorylation was inhibited at all time points analyzed, indicating the effectiveness of AG1478. The transient increase in the phosphorylation of AKT and ERKs following EGF stimulation was also inhibited by treatment with AG1478. EGF stimulation caused a transient reduction in the basal level of phosphorylated STAT3^Tyr705^ at 1 h in three of four cell lines, however, STAT3^Tyr705^ phosphorylation returned to basal levels by 18 h. However, AG1478 treatment did not inhibit the constitutive STAT3^Tyr705^ phosphorylation in EGF stimulated cells (Figure 2B). Treating cells with AG1478 blocked the transient reduction of phosphorylated STAT3^Tyr705^ following EGF induction. This suggests the possibilities that EGFR signaling may induce the activation of a specific phosphatase or cause an increase in the turnover of phosphorylated form of STAT3^Tyr705^. More pertinent to the current study, these observations suggest that constitutive STAT3^Tyr705^ phosphorylation does not require EGFR signaling in PDAC cells. However, inhibiting EGFR activation with AG1478 affects other known down-stream signaling molecules including phosphorylation of AKT and ERKs (Figure 2B) thus proving the efficacy of inhibiting EGFR by AG1478 in the cell lines tested.

### Combination of AG1478 and gemcitabine does not cause synergistic growth inhibition of PDAC cells *in vitro* and does not block constitutive STAT3^Tyr705^ phosphorylation

**Figure 3:**
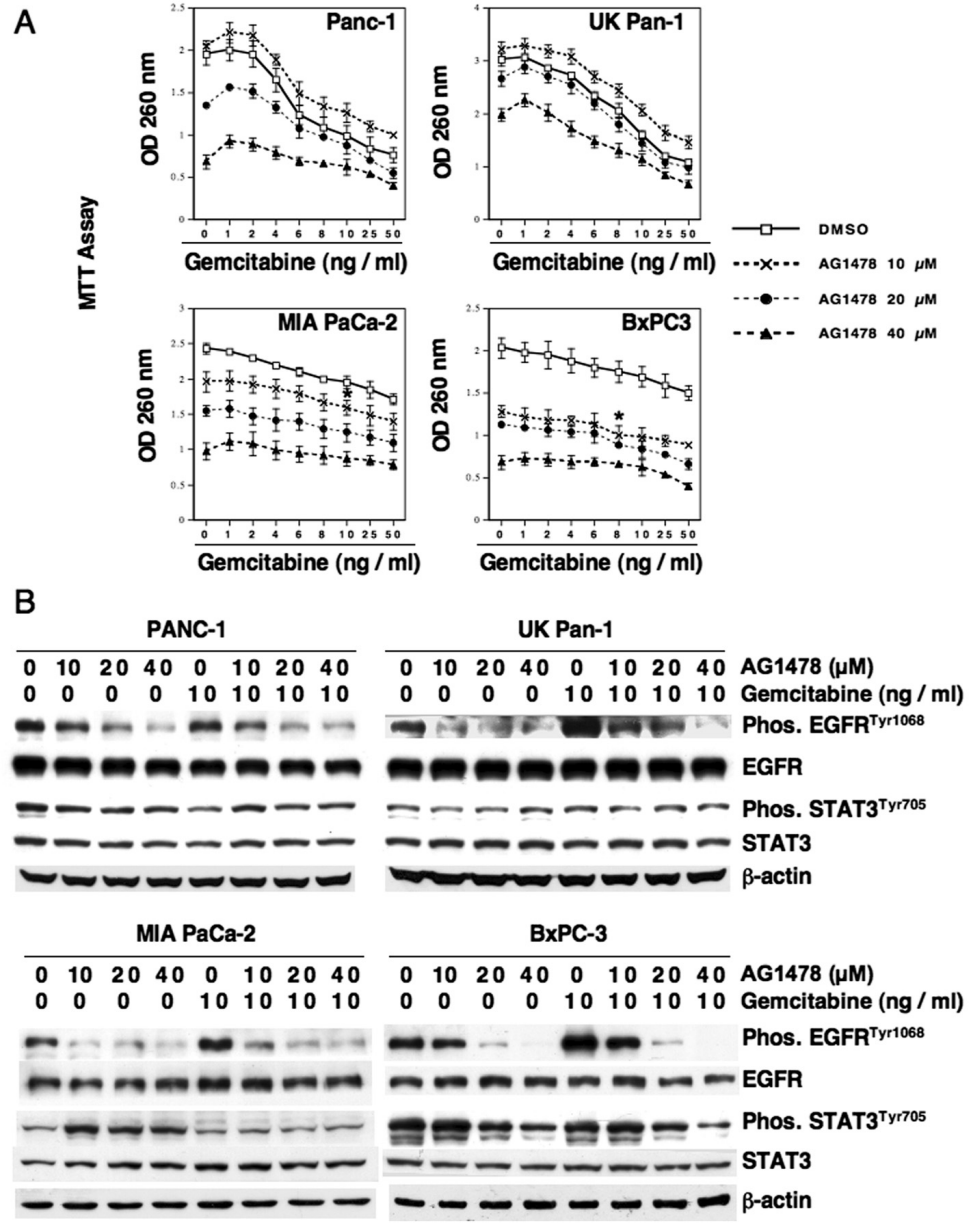
**Combination of AG1478 and gemcitabine does not cause synergistic growth inhibition and does not prevent constitutive STAT3**^**Tyr705 **^**phosphorylation of PDAC cells. A**, Exponentially growing PDAC cells were exposed to indicated concentrations of EGFR inhibitor, AG1478, gemcitabine or both together. MTT assays were performed to measure growth inhibition after 96 h of treatment. Bars represent SD of eight replicates, and the experiments were repeated three times. (*) = significant growth inhibition (p < 0.001) starts from this dose level and remained significant at higher doses. **B**, Exponentially growing PDAC cells were exposed to indicated concentrations of AG1478, gemcitabine (10 ng/ml), or both together. The blots were probed for phosphorylated and total forms of EGFR and STAT3. As a loading control, human β-actin levels are shown.

Treatment with gemcitabine is reported to activate EGFR [20] and therefore targeting EGFR might be expected to mitigate pro-survival signaling induced by this pathway [19]. We next determined the combined effect of AG1478 and gemcitabine on the growth of PDAC cell lines *in vitro*. Cells were treated with AG1478 and gemcitabine separately or in combination. Rates of growth were assessed by MTT assays following 96 h of treatment and a representative data is shown in Figure 3A. For MIA PaCA-2 and BxPC3 cells, a significant increase in growth inhibition was observed for combined therapy at the lowest concentration of AG1478 used (10 μM) and required concentrations of gemcitabine of at least 8 ng/ml (Figure 3A). PANC-1 cells showed an increase of growth inhibition by gemcitabine when only combined with 20 and 40 μM dose of AG1478; however when compared to gemcitabine treatment alone, the growth inhibition achieved by combining both agents was only incremental. In UK Pan-1 cells, a significant effect was observed for combined treatment when the highest concentration of AG1478 (40 μM) was used in combination and as seen with PANC-1 cells, the combination treatment caused only a marginal increase of growth suppression (Figure 3A). These observations suggest, although the combined treatments increased growth inhibition, the effects were less than additive.

STAT3^Tyr705^ phosphorylation was not inhibited by treating cells with either AG1478 or gemcitabine alone, except in BxPC3, where higher concentrations of AG1478 (40 μM) caused some inhibition (Figure 3B). Similarly, combining both drugs had a minimal affect on the level of STAT3^Tyr705^ phosphorylation except for BxPC3 where higher doses of AG1478 (>20 μM) resulted in some reduction of STAT3^Tyr705^ phosphorylation (Figure 3B). It should be noted that 10 μM concentration of AG1478 was sufficient to inhibit phosphorylation of EGFR suggesting that molecular affects requiring concentrations of AG1478 greater than 10 μM may represent off-target effects.

### Inhibition of STAT3 by shRNA sensitizes PDAC cells to gemcitabine *in vitro*

**Figure 4:**
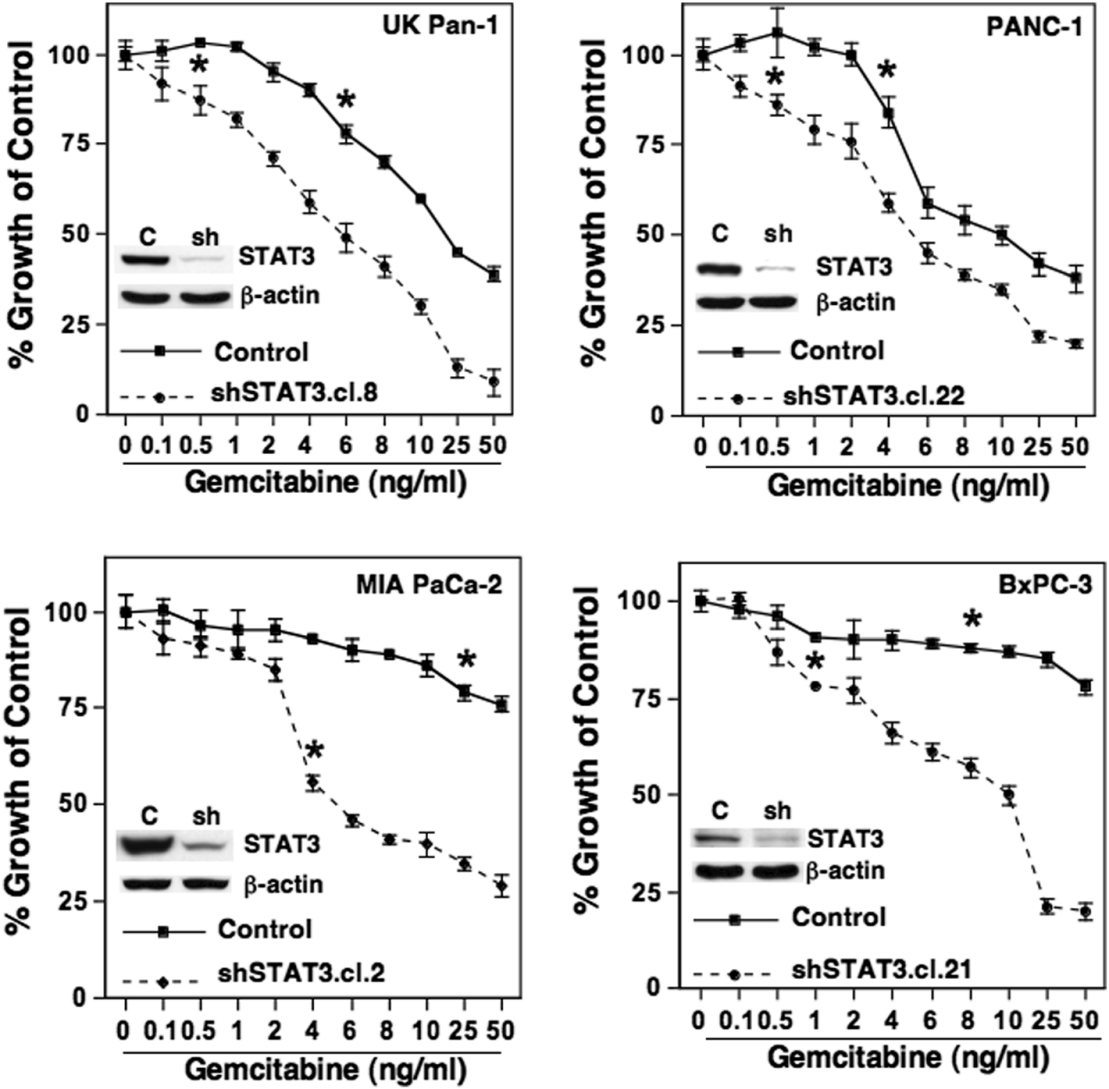
**Inhibition of STAT3 by shRNA sensitizes PDAC cells to Gemcitabine *in vitro*.** PDAC cells and their respective STAT3-shRNA clone cells were treated with indicated concentrations of gemcitabine for 96 h and MTT assays were performed to measure the rate of growth. Data are presented as % growth of control. Bars represent SD of eight replicates, repeated three times. (*) = significant growth inhibition (p < 0.001) starts from this dose point and remains significant at higher dose levels. Insets: Western blots showing differences in the expression of STAT3 in control and shSTAT3 clone cells. As a loading control, β-actin levels are shown.

Because STAT3^Tyr705^ phosphorylation was maintained in cells treated with AG1478 or gemcitabine, we hypothesized that targeting STAT3 may serve as an independent therapeutic target or may cause PDAC cells to be more sensitive to gemcitabine. To inhibit STAT3, PDAC cells PANC-1, UK Pan-1, MIA PaCa-2 and BxPC3 were transfected with a vector that expresses a shRNA against STAT3 and individual stable clones were established after antibiotic selection. These clones were tested for the expression of STAT3 (data not shown) along with control cells that express the vector alone. Control cells and isogenically matched cells that express STAT3-shRNA were treated with gemcitabine and were assessed for growth by MTT assays. As shown in Figure 4, cells that express shRNA against STAT3 were significantly more sensitive to gemcitabine treatment as compared to control cells. UK Pan-1 and PANC-1 cells showed a significant dose dependent sensitivity to gemcitabine at doses of 6 and 4 ng/ml respectively and knockdown of STAT3 further increased their sensitivity as significant growth inhibition was observed from 0.5 ng/ml and greater. MIA PaCa-2 and BxPC3 cells were more resistant to gemcitabine compared to UK Pan-1 and PANC-1 (Figure 4). Statistically significant growth inhibition was observed for doses of gemcitabine from 25 ng/ml and above for MIA PaCa-2 cells and 8 ng/ml and greater for BxPC3 cells. Interestingly, knockdown of STAT3 increased their sensitivity to gemcitabine to a level similar to that seen for the more sensitive cell lines, UK Pan-1 and PANC-1 (Figure 4). Significant growth inhibition was seen in STAT3 knock down cells at doses of 4 ng/ml and 1 ng/ml for MIA PaCa-2 and BxPC3 cells respectively. The relative expression levels of STAT3 as determined by Western blot analyses are shown as insets within the graph for the respective cell lines along with β-actin as a loading control. We further evaluated whether knocking down STAT3 sensitizes the cells to EGFR inhibitor, AG1478. However, AG1478 treatment of STAT3 knockdown cells did not cause a significant increase in growth inhibition above that seen with control cells (Additional file 1: Figure S1). This result suggests that targeting STAT3 enhances response to gemcitabine-mediated growth suppression, but not to the EGFR kinase inhibitor in the cell lines tested. Conversely, over expressing STAT3 in PANC-1 cells, caused these cells to be less sensitive to gemcitabine induced growth inhibition. Vector transfected control cells showed a significant growth inhibition at a dose of 4 ng/ml; whereas, the STAT3 over expressing PANC-1 cells required a two fold increase in the amount of gemcitabine (8 ng/ml) for significant growth inhibition (Additional file 2: Figure S2). This finding further supports the results of the knock down experiments indicating that STAT3 plays a role in reducing the response of PDAC cells to gemcitabine.

### Increased sensitivity to gemcitabine in STAT3 shRNA cells is mediated by the induction of apoptosis and growth arrest

Human PDAC cells that initially respond to gemcitabine frequently develop resistance to treatment [21,22]. Different signaling pathways contribute to resistance against apoptosis in pancreatic cancer cells [23]. Previous studies indicate that mitochondria mediated apoptosis is important for gemcitabine sensitivity. STAT3 is known to promote anti-apoptotic signals in many cancer types [24]. Because sensitivity to gemcitabine was enhanced in cells where STAT3 was knocked down, we next tested whether increased growth inhibition was accompanied with induction of apoptotic signaling. Control and STAT3-shRNA expressing cells were treated with gemcitabine for 96 h and then analyzed for caspase-3 activity by flow cytometry. In control cells, gemcitabine treatment did not show considerable caspase-3 activity, suggesting that they are refractory to gemcitabine-mediated apoptosis at the concentrations used in this study. STAT3 knockdown cells showed an appreciable increase in caspase-3 activity upon treatment with gemcitabine (Table 1). However, knockdown of STAT3 did not cause as much apoptosis in the MIA PaCa-2 and BxPC3 cells treated with gemcitabine (10.0% and 5.7% for 25 ng/mL of gemcitabine) compared to the PANC-1 and UK Pan-1 cells (55.4% and 35.6% for 25 ng/mL of gemcitabine); (Table 1). This suggests that the enhanced response to gemcitabine seen in MIA PaCa-2 and BxPC3 cells is caused by a combination of growth arrest and apoptosis. To address this possibility, cell cycle analysis was performed in control and shSTAT3 knockdown cells of MIA PaCa-2 and BxPC3 cells. Interestingly, G1 arrest in shSTAT3 knockdown cells was greater after treatment with gemcitabine. In MIA PaCa-2/shSTAT3 cells, the percentage of cells at G1 phase was 47.5%, and treatment with gemcitabine increased the levels to 70.3%. Similarly in BxPC3/shSTAT3 cells treatment with gemcitabine increased the percentage of cells in G1 phase to 70% as compared to untreated cells showing only 38.2% cells. The G1 phase in the MIA PaCa-2 and BxPC3 vector control cells was not appreciably affected by treatment with gemcitabine (Table 2).

**Table 1:** Percent apoptotic cells as measured by Caspase-3 activity in cells treated with gemcitabine

	**% apoptotic cells**		
	**Gemcitabine (ng/ml)**		
	**0**	**10**	**25**
UK Pan-1/Control	0.38	0.97	3.14
UK Pan-1/shSTAT3.cl.8	3.1	24.4	35.6
PANC-1/Control	0.01	0.01	.02
PANC-1/shSTAT3.cl.22	4.7	6.0	55.4
MIA PaCa-2/Control	0.06	0.06	0.07
MIA PaCa-2/shSTAT3.cl.2	2.6	5.5	10.0
BxPC3/Control	0.04	0.17	0.12
BxPC3/shSTAT3.cl.21	0.4	1.6	5.7

**Table 2:** Cell cycle analysis of cells treated with gemcitabine

	**% of cells at various phases of cell cycle**					
**Cell line**	**Untreated**			**Gemcitabine (10 ng/ml)**		
	**G1**	**S**	**G2**	**G1**	**S**	**G2**
MIA PaCa-2/Control	62.1	21.4	15.7	56.1	24.3	18.8
MIA PaCa-2/shSTAT3	47.5	32.9	17.8	70.3	13.9	14.9
BxPC3/Control	32.5	45.3	19.8	31.8	44.3	21.8
BxPC3/shSTAT3	38.2	33.8	25.1	70.0	15.0	13.6

### Inhibition of STAT3 by shRNA suppressed the growth of tumors *in vivo* and increased sensitivity to gemcitabine

**Figure 5:**
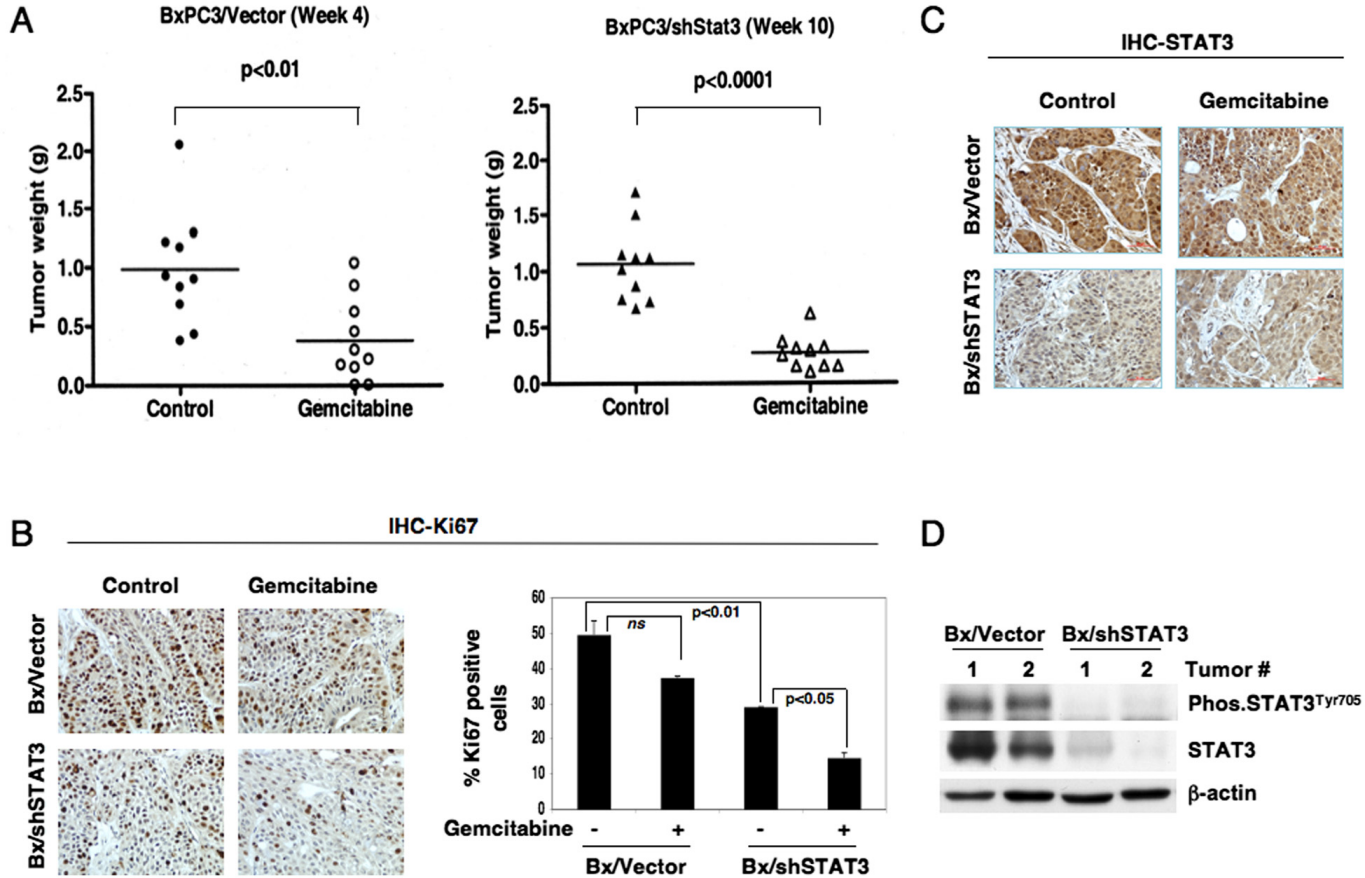
**Inhibition of STAT3 suppressed the growth of tumors *in vivo*. A**, BxPC3/Vector and shSTAT3 expressing (BxPC3/shSTAT3) cells were orthotopically implanted in a mouse model. Both group of animals were treated with gemcitabine (20 mg/kg body weight) or saline IP every three days until sacrifice. Primary tumors were surgically removed at week 4 and week 10 respectively for BxPC3/Vector and BxPC3/shSTAT3 group of animals. The isolated tumors were weighed to measure the growth of tumors and a representative data is shown for 10 animals in each group. Formalin fixed tumor tissue sections were analyzed for nuclear Ki-67 **(B)** and STAT3 **(C)** by immunohistochemistry. **D**, Total cellular proteins isolated from the tumors of BxPC3/Vector and BxPC3/shSTAT3 group of animals were analyzed by Western blot for the expression of STAT3^Tyr705^. β-actin and STAT3 are shown as a loading control.

To further validate the data observed *in vitro*, an orthotopic mouse pancreatic cancer model was utilized [25] to assess STAT3 as a target for therapy *in vivo*. Control BxPC3/Vector cells and isogenically matched BxPC3 cells expressing shSTAT3 (BxPC3/shSTAT3) were implanted orthotopically. Tumors derived from mice implanted with control BxPC3/Vector cells developed rapidly and were measured four weeks after implantation (Figure 5A); whereas, mice implanted with BxPC3/shSTAT3 cells showed a delay in tumor development and therefore tumors in these animals were allowed to grow until week ten. Treatment with gemcitabine significantly (p < 0.0001) reduced the growth of tumors from BxPC3/shSTAT3 group of animals as compared to control group of animals treated with gemcitabine. These experiments were repeated several times although with a fewer number of animals. The observations were similar in all the repeat experiments, i.e., the control (BxPC3/Vector) group of animals always formed large palpable tumors between weeks four and six. Tumor growth was delayed in mice implanted with BxPC3/shSTAT3 cells by an additional 4–6 weeks compared to BxPC3/Vector (data not shown for repeat experiments).

Tumor tissues were further analyzed by immunohistochemistry (IHC) for STAT3 and Ki-67. Nuclear expression of Ki-67 was used as a marker for proliferation and STAT3 staining was used to confirm that STAT3 was knocked down in tumors from the BxPC3/shSTAT3 group. Tumors in the control group (BxPC3/Vector) showed 49.5% Ki-67 positive cells and treatment with gemcitabine reduced the expression level of Ki-67 to 37.3% (Figure 5B). In tumors derived from the mice implanted with BxPC3/shSTAT3 cells, nuclear expression of Ki-67 was significantly (p < 0.01) reduced to 29.0% as compared to 49.5% for BxPC3/Vector group. Treatment with gemcitabine further and significantly (p < 0.05) reduced the levels to 14.6% in the STAT3 knockdown group (Figure 5B). As expected, tumors derived from BxPC3/shSTAT3 group of animals showed reduced expression of STAT3 (Figure 5C) as determined by immunohistochemistry. Total cellular proteins were isolated from the tumors of both groups (BxPC3/Vector and BxPC3/shSTAT3) and subjected to Western blot analysis to assess the levels of both phosphorylated and total forms of STAT3. Consistent to the observations made from immunohistochemistry, tumors from BxPC3/shSTAT3 showed diminished levels of STAT3. Similar to STAT3, the phosphorylated levels of STAT3^Tyr705^ were also reduced as shown in the Western blot and as a loading control β-actin are shown (Figure 5D).

## Discussion

Treatment with gemcitabine continues to be the standard mode of therapy either as a single agent or in combination with an EGFR inhibitor; however, PDAC still remains a great challenge in oncology as the rate of mortality nears the rate of incidence [1]. In this study, we sought to identify pro-survival pathways that are activated in the presence of gemcitabine and an EGFR inhibitor, AG1478, using PDAC cell line models.

Interestingly, STAT3^Tyr705^ phosphorylation was not inhibited by treatment with AG1478 except for, a partial inhibition that was observed in BxPC3 cells treated for 96 h with higher concentrations of AG1478. STAT3^Tyr705^ phosphorylation is considered to be a down stream target of EGFR signaling in some cell types [26]. However, other studies showed that inhibiting EGFR signaling did not affect STAT3^Tyr705^ phosphorylation [27-29]. Skin biopsies of patients treated with the EGFR inhibitor Gefitinib showed a decreased EGFR activation that was associated with an increase in STAT3^Tyr705^ phosphorylation [28]. In a majority of the HNSCC cells lines tested, inhibition of EGFR signaling by AG1478 did not affect the overall STAT3^Tyr705^ phosphorylation levels, while EGFR, ERKs and STAT3^Ser727^ phosphorylation was inhibited [29]. In agreement with these latter studies, the data presented here indicates that constitutive STAT3^Tyr705^ phosphorylation does not require EGFR signaling in the four human PDAC cell lines that were examined. As anticipated, treatment with AG1478 of the four PDAC cell lines used in this study did show inhibition of phosphorylation of EGFR, AKT and ERKs (Figure 2B). Thus the growth suppressive effect of AG1478 may be attributable to a reduction of the phosphorylation of AKT or ERKs, which are also known to play a role in tumor progression. However, even after effective inhibition of EGFR signaling, the presence of constitutive STAT3^Tyr705^ phosphorylation may decrease the response to chemotherapy by inducing pro-survival pathways. Similar to this observation, treatment of cells with gemcitabine either alone (Figure 1) or in combination with AG1478 did not affect the constitutive STAT3^Tyr705^ phosphorylation (Figure 3B). The presence of constitutive phosphorylation of STAT3^Tyr705^ following treatment with AG1478 or gemcitabine prompted us to investigate whether inhibiting STAT3 would increase the sensitivity of PDAC cells to chemotherapy.

Interestingly, PDAC cells with knockdown of STAT3 demonstrated a similar exponential growth rate as the control cells *in vitro*. However, PDAC cells with STAT3 knocked down showed a decreased colony forming ability when plated at low density suggesting a reduced oncogenic phenotype (data not shown). Cells where STAT3 was knocked down showed a significant increase of growth inhibitory response to gemcitabine (Figure 4). STAT3 knockdowns of PANC-1 and UK Pan-1 cells showed significant growth inhibition from 0.5 ng/ml dose of gemcitabine as compared to 4 and 6 ng/ml of gemcitabine required to cause significant growth inhibition of their respective control cells. BxPC3 and MIA PaCa-2 cells showed a greater resistance to gemcitabine compared to PANC-1 and UK Pan-1. Knockdown of STAT3 in the gemcitabine resistant PDAC cell lines (BxPC3 and MIA PaCa-2) resulted in a significant increase of growth suppression. Control MIA PaCa-2 and BxPC3 cells required 25 and 8 ng/ml of gemcitabine respectively to inhibit growth significantly; whereas 4 and 1 ng/ml of gemcitabine was needed to cause significant growth inhibition in cells where STAT3 was knocked down (Figure 4). The response of BxPC3 and MIA PaCa-2 cells where STAT3 was knocked down was comparable to the control group of PANC-1 and UK Pan-1 cells. In addition, the sensitivity to gemcitabine achieved by knocking down STAT3 was much greater than that observed by combining AG1478 and gemcitabine. It is interesting that cell lines PANC-1 and UK Pan-1 possess intact TGF-β signaling components while cell lines BxPC3 and MIA PaCa-2 lack TGF-β signaling due to lack of Smad4 or because of transcriptional repression of TGF-β type II receptor, respectively [30]. We previously observed that restoration of Smad4 in PDAC cells suppressed the levels of STAT3^Tyr705^ phosphorylation and reversed the TGF-β mediated invasion [25]. Additional studies are needed to determine whether inhibiting STAT3 may be of further therapeutic benefit in cells that lack intact TGF-β signaling.

Over-expression of STAT3 reduced the gemcitabine induced growth suppression in PANC-1 cells (Additional file 2: Figure S2). This observation further supporting the notion that STAT3 play a role in mediating reduced sensitivity to gemcitabine of PDAC cells.

A recent study [18] showed that suppression of RON sensitized PDAC cells to gemcitabine. The observations from this study showed PDAC cells used in this study expressed varying levels of RON expression, but treatment with gemcitabine did not appreciably alter RON levels (Figure 1). However, inhibition of STAT3 in these PDAC cells did sensitize them to gemcitabine. Thus, inhibiting STAT3 in high RON expressing cells may provide a novel approach for enhancing tumor response to gemcitabine.

Human PDAC cells are known to have inherent resistance or to develop resistance against gemcitabine mediated apoptosis [31]. Treatment with gemcitabine did not induce considerable pro-apoptotic signals in the cell lines tested in this study. However, STAT3 knockdown in PANC-1 and UK Pan caused a dramatic increase in caspase-3 activity. Whereas, in MIA PaCa-2 and BxPC3 cells, knockdown of STAT3 resulted in only a modest increase of caspase-3 activity upon treatment with gemcitabine, but was accompanied with an increase in G1 cell cycle arrest (Tables 1 and 2).

While knockdown of STAT3 rendered PDAC cells sensitive to gemcitabine mediated killing, these cells did not show enhanced growth suppression when treated with EGFR inhibitor AG1478. Further studies are needed to verify what other targets are responsible for this phenomena.

To further validate these *in vitro* findings, mice were orthotopically implanted with BxPC3 control cells or with the isogenically matched BxPC3/shSTAT3 cells. Mice implanted with control cells and treated with saline had large tumors by week four. Mice implanted with control cells and treated with gemcitabine had smaller tumors at this point, confirming that these tumors responded to gemcitabine *in vivo*. However, mice implanted with Bx/shSTAT3 cells did not show palpable tumors by week four; tumors similar in size to the control group did not develop until week ten. Treatment with gemcitabine resulted in significantly smaller tumors in mice implanted with shSTAT3 cells indicating that a combination of gemcitabine and knockdown of STAT3 results in a significant reduction of tumor growth over either one alone. A multitude of signaling events by STAT3 may converge to enhance tumor progression with increased resistance against chemotherapeutic agents. The findings of this study suggest that constitutive STAT3^Tyr705^ activation may play an important role in pancreatic oncogenesis that is independent of EGFR signaling and thus may be an important biologic target. Moreover, these data suggest that targeting STAT3 may increase response to gemcitabine and may reverse, at least in part, resistance to this chemotherapeutic agent. Currently there are great efforts to develop clinically relevant inhibitors for STAT3 [14,32] and thus these new agents should be tested, as they become available, in combination with current standard chemotherapy.

## Conclusions

The observations of this study demonstrate that oncogenic constitutive STAT3^Tyr705^ phosphorylation is not affected by treatment of PDAC cells with gemcitabine or AG1478 either alone or in combination. Both the agents together did not induce synergistic growth inhibition suggesting that STAT3 may be a target to enhance the overall response to chemotherapy. Knockdown of STAT3 in PDAC cells enhanced their response to gemcitabine mediated cell growth inhibition in part due to increased pro-apoptotic activity as evidenced by an induction of caspase-3 activity or an increase of G1 cell cycle arrest. However, knockdown of STAT3 did not enhance the growth suppressive activity of an EGFR inhibitor, AG1478. *In vivo* orthotopic animal studies further confirmed that STAT3 could be a viable target in PDAC cells to increase the sensitivity to gemcitabine. Knocking-down STAT3 significantly reduced the tumor burden as evidenced by a slower tumor progression and further reduced the growth of tumors that is associated with a reduction of Ki-67 positive cells. This study suggests that STAT3 is to be considered a viable target to enhance chemotherapeutic response of PDAC cells.

## Methods

### Cell lines

Established human PDAC cell lines PANC-1, BxPC3 and MIA PaCa-2 used in this study were purchased from American Type Culture Collection (Manassas, VA). UK Pan-1 cell line was established in our laboratory [33]. Cell lines (PANC-1, UK Pan-1 and MIA PaCa-2) were grown in DMEM (Cell Grow-Mediatech) and BxPC3 cells were grown in RPMI medium (InVitrogen). Both types of media were supplemented with 10% fetal bovine serum (Cell Grow-Mediatech).

### Reagents

Commercially available EGFR inhibitor AG1478 was purchased from EMD Biosciences and gemcitabine was purchased from LTK Corporation. AG1478 was solubilized in DMSO and gemcitabine was dissolved in PBS. For animal injections, pharmaceutical grade gemcitabine (200 mg vial for injection) was used (Dr. Reddy’s Laboratories, Limited, India).

### Cell growth assays

The growth rate of AG1478 or gemcitabine treated cells was determined by 3-(4,5-dimethylthiazol-2-yl)-2,5-diphenyltetrazolium bromide (MTT) assays as described previously [30]. Exponentially growing cells (1–2 × 10^3^) were plated in 96-well plates. Cells were treated with indicated concentrations of either gemcitabine or AG1478 or treated with both agents in combination. MTT assays were performed after 96 h of treatment. At the end of treatment period, cells were stained with 0.5 mg/mL MTT (Sigma Chemical Company) at 37°C for 2 h. MTT containing medium was aspirated and the cells were solubilized in 200 μL of DMSO. Colorimetric determination was done with a Molecular Devices plate reader. The data are represented as the mean value of eight wells per treatment group and the experiments were repeated a minimum of three times. To evaluate differences between treatment groups, analysis of variance (ANOVA) combined with Tukey’s multiple range test was performed (GraphPad Software, Inc.) and considered statistically significant when p < 0.001.

### Stable transfections

To knockdown STAT3, cells were transfected with Sure Silencing shRNA-STAT3 plasmid (SuperArray Bioscience Corporation) according to manufacturer’s suggestion using FuGene 6 (GE) transfection reagent as previously reported [25]. Cells were cultured further and selected in medium containing 620 μg/mL G418 for PANC-1, UK Pan-1 and MIA PaCa-2 cells or 200 μg/mL G418 for BxPC3 cells. Individual G418-resistant colonies were isolated during drug selection and established as individual clones for further analysis. To over-express STAT3, PANC-1 cells were transfected with STAT3 cDNA (a kind gift from Dr. Richard Jove’s lab) using FuGene 6 (GE) and G418-resistant clones were isolated and established as individual clones for further studies.

### Western immunoblots

Total cellular proteins were extracted by using Laemmli buffer and Western immunoblots were done as described previously [30]. Cells were harvested at indicated time points after treatment with AG1478 or gemcitabine along with appropriate controls. Seventy micrograms of protein lysates were electrophoresed on an 8% SDS-polyacrylamide gel and then transferred to Hybond-P, polyvinylidene difluoride membrane (BioRad). Primary antibodies for EGFR, β-actin, STAT3, phosphorylated STAT3^Tyr705^ and RON were from Santa Cruz Biotechnology; phosphorylated EGFR^Tyr1068^ ERKs, phosphorylated ERKs^Thr202/Tyr204^, AKT, phosphorylated AKT^Ser473^ were from Cell Signaling Technology. The blots were probed with specific antibodies and detected by enhanced chemiluminescence methods (Pierce).

### Active Caspase-3 assay and cell cycle analysis by flow cytometry

Cells undergoing apoptosis was determined by flow cytometry using a BD Pharmingen active Caspase-3 FITC kit. Cells were treated with indicated concentrations of gemcitabine for 96 h and processed for flow cytometric analysis per manufacturer’s suggestion. Data are presented as the percent of apoptotic cells. Exponentially growing MIA PaCa-2 and BxPC3 cells were treated with gemcitabine (10 ng/mL) for 24 h and cell cycle analysis was performed by flow cytometry. The above mentioned flow cytometry experiments were conducted with FACSCalibur (Becton Dickinson Immunocytometry Systems Inc, San Jose) at our institutional core flow cytometry facility.

### Orthotopic pancreatic cancer mouse model

4–5 week-old athymic nude mice were purchased from Harlan Corp. Mice were housed and maintained in accordance with the standards of The University of Texas Health Science Center at San Antonio Animal Care and Use Committee. BxPC3/Vector or BxPC3/shSTAT3 cells were grown to 80% confluence, trypsinized and re-suspended in PBS, and then 1 × 10^6^ cells/50 μL were injected directly into the pancreas of anesthetized mice [25]. Two weeks after implantation, mice were injected with either gemcitabine (20 mg/kg) or saline IP every 3 days until sacrifice. Primary tumors were surgically removed and weighed. Statistical analysis was determined by t-test (GraphPad Software, Inc.). Statistical significance between control and gemcitabine treated groups was considered when p < 0.05.

### Immunohistochemistry

Tumors derived from control and experimental groups were fixed in formalin. The paraffin embedded tumor tissue sections were processed at our institutional core facility, Histology and Pathology Laboratory at the University of Texas Health Science Center at San Antonio for the expression of Ki-67 and STAT3 per standard procedures. Two tumors were analyzed from each treatment group for the analysis of Ki-67 positive staining. Digital image analysis was carried out to determine the nuclear staining levels by using “ImmunoRatio”, a web based analysis software [34]. A minimum of ten microscopic field areas were analyzed for each tumor slide and the data were plotted as percent positive cells for Ki-67 staining. Statistical significance (p < 0.05) was evaluated by the ANOVA combined with Tukey’s multiple range test (GraphPad Software, Inc.).

## Competing interests

The authors declared that they have no competing interests.

## Authors’ contributions

KV and JWF developed the concept and design of the project. KV, LP and JWF wrote and edited the manuscript. KV, LP, SZ, STN and JWF analyzed and interpreted data. LP, SZ and STN substantially contributed for the *in vivo *animal model studies. KV and SZ conducted flow cytometric assays. PH, LC and QZ provided general technical support for Western blots, MTT assays and animal studies. All authors read and approved the final manuscript.

## Supplementary Material

Additional file 1: Figure S1 AG1478 induced growth inhibition of PDAC cells is not altered by knocking down STAT3. Exponentially growing PDAC cells and their respective shSTAT3 clones were treated with the indicated concentrations of EGFR inhibitor, AG1478. MTT assays were performed to measure growth after 96 h of treatment. Click here for file

Additional file 2: Figure S2 STAT3 over-expression decreases gemcitabine mediated growth inhibition of PANC-1 cells. PANC-1 control cells expressing an empty vector (C) or PANC-1 cells expressing STAT3 cDNA (OE) cells were treated with indicated concentrations of gemcitabine for 96 h and MTT assays were performed to analyze the growth. *, Significant growth inhibition (p < 0.001) starts from this dose point and beyond. Inset: Western blot showing the over-expression of STAT3 as compared with control cells. Human β-actin is used as a loading control. Click here for file
