# Supplementary figures and images for: Inhibiting signal transducer and activator of transcription-3 increases response to gemcitabine and delays progression of pancreatic cancer
Source: Mol Cancer. 2013 Sep 11;12:104. doi: 10.1186/1476-4598-12-104 (PMC3847497; doi:10.1186/1476-4598-12-104)

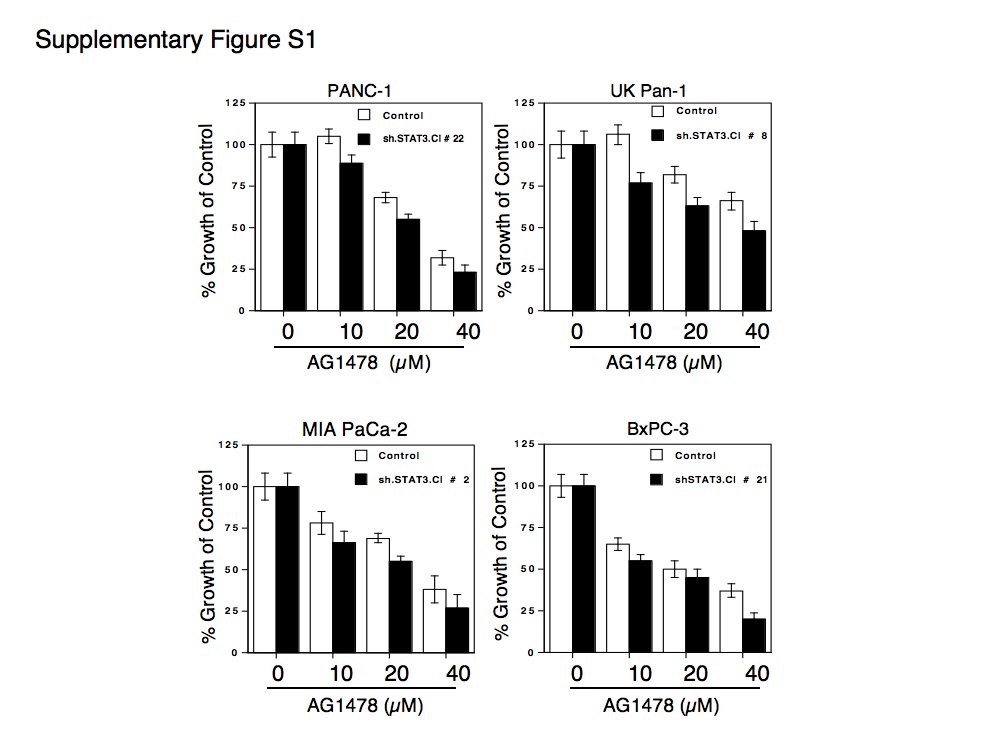

Supplement: Additional file 1: Figure S1 — AG1478 induced growth inhibition of PDAC cells is not altered by knocking down STAT3. Exponentially growing PDAC cells and their respective shSTAT3 clones were treated with the indicated concentrations of EGFR inhibitor, AG1478. MTT assays were performed to measure growth after 96 h of treatment. [file 1476-4598-12-104-S1.tiff]

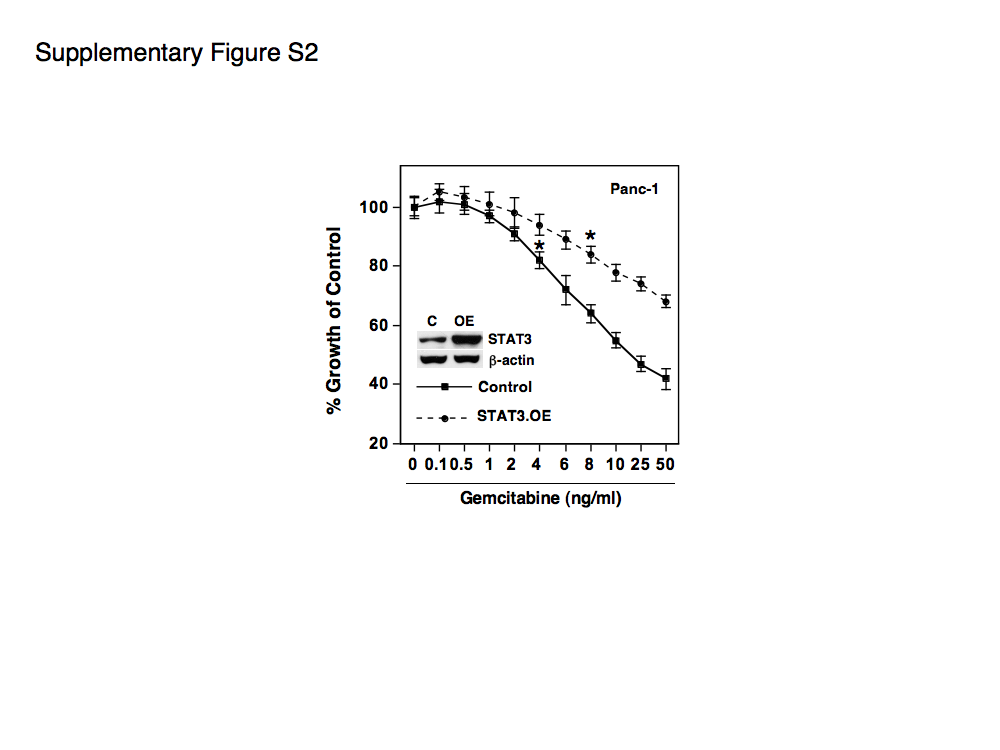

Supplement: Additional file 2: Figure S2 — STAT3 over-expression decreases gemcitabine mediated growth inhibition of PANC-1 cells. PANC-1 control cells expressing an empty vector (C) or PANC-1 cells expressing STAT3 cDNA (OE) cells were treated with indicated concentrations of gemcitabine for 96 h and MTT assays were performed to analyze the growth. *, Significant growth inhibition (p < 0.001) starts from this dose point and beyond. Inset: Western blot showing the over-expression of STAT3 as compared with control cells. Human β-actin is used as a loading control. [file 1476-4598-12-104-S2.tiff]
